# Residual Stresses in Metal Manufacturing: A Bibliometric Review

**DOI:** 10.3390/ma18153612

**Published:** 2025-07-31

**Authors:** Diego Vergara, Pablo Fernández-Arias, Edwan Anderson Ariza-Echeverri, Antonio del Bosque

**Affiliations:** 1 Technology, Instruction and Design in Engineering and Education Research Group (TiDEE.rg), Catholic University of Avila, C/Canteros s/n, 05005 Ávila, Spain; pablo.fernandezarias@ucavila.es (P.F.-A.); antonio.bosque@ucavila.es (A.d.B.); 2 Facultad de Ingeniería, Universidad del Magdalena, Santa Marta 470004, Colombia; earizaec@unimagdalena.edu.co

**Keywords:** residual stress, metal, manufacturing, microstructure, mechanical, bibliometric review

## Abstract

The growing complexity of modern manufacturing has intensified the need for precise control of residual stresses to ensure structural reliability, dimensional stability, and material performance. This study conducts a bibliometric review using data from Scopus and Web of Science, covering publications from 2019 to 2024. Residual stress research in metal manufacturing has gained prominence, particularly in relation to welding, additive manufacturing, and machining—processes that induce significant stress gradients affecting mechanical behavior and service life. Emerging trends focus on simulation-based prediction methods, such as the finite element method, heat treatment optimization, and stress-induced defect prevention. Key thematic clusters include process-induced microstructural changes, mechanical property enhancement, and the integration of modeling with experimental validation. By analyzing the evolution of research output, global collaboration networks, and process-specific contributions, this review provides a comprehensive overview of current challenges and identifies strategic directions for future research in residual stress management in advanced metal manufacturing.

## 1. Introduction

### 1.1. Residual Stresses in Metal Manufacturing

In the field of materials science and engineering, the reliability and performance of metallic components are critically influenced by residual stresses, which are self-equilibrating internal stresses that remain within a material after manufacturing, even when no external forces are applied [1,2]. These “locked-in” stresses are an almost unavoidable consequence of processes that induce non-uniform inelastic (plastic) deformation, originating from sources such as severe thermal gradients during rapid heating and cooling, mechanical operations, or solid-state phase transformations [3,4]. The impact of residual stress on a component’s integrity is a classic duality: it can be either highly beneficial or severely detrimental. Compressive residual stresses, particularly at the surface, are generally desirable as they can significantly enhance a component’s service life by increasing its resistance to failure mechanisms like fatigue and stress corrosion cracking [5]. By opposing the tensile stresses applied during service, they effectively raise the threshold required to initiate and propagate cracks. Conversely, tensile residual stresses are typically harmful, as they can substantially decrease fatigue life, reduce fracture toughness, and promote premature failure by lowering the effective strength of the material. In processes like metal additive manufacturing, high tensile stresses can lead to significant part distortion, dimensional instability, and even catastrophic cracking or debonding from the build plate during fabrication [6]. Therefore, understanding, predicting, and controlling these internal stresses is a fundamental challenge in modern manufacturing to ensure the structural reliability and performance of finished metal parts.

Virtually all manufacturing processes introduce some level of residual stress, as they inherently involve non-uniform plastic deformation, thermal cycles, or phase changes [7,8]. These stresses are not confined to a single type of operation but are a common feature across a wide range of fabrication methods. Welding, for example, is a primary source of significant tensile residual stresses due to the intense localized heating followed by rapid cooling, which creates incompatible thermal strains that the surrounding cooler material must accommodate [9,10]. Similarly, additive manufacturing (AM) processes, such as powder bed fusion, are notorious for generating high-magnitude residual stresses due to the extremely large and localized thermal gradients that occur as material is melted and rapidly solidifies layer by layer [8,11]. Subtractive processes like machining introduce complex stress profiles through the combined action of mechanical plastic deformation from the cutting tool and thermal gradients generated by friction at the tool–workpiece interface [12,13]. Likewise, metal-forming operations impart stresses through extensive plastic deformation [14,15,16]; processes like cold drawing, for instance, typically create a characteristic stress profile with tensile stresses in the core and beneficial compressive stresses at the surface [17,18]. Casting also introduces residual stress as the molten metal cools and solidifies under the geometric constraints of the mold, leading to differential cooling rates and internal strain [19]. The complexity is further amplified in hybrid manufacturing techniques, like extrusion bonding, where stresses can arise from a combination of both severe plastic deformation and the thermal expansion mismatch between different materials [20]. Furthermore, heat treatments like quenching can generate high internal stresses, particularly in steels, due to volume changes associated with phase transformations, such as the formation of martensite [21]. It is also critical to recognize that subsequent operations can alter existing stress fields; for instance, milling or cutting a previously welded component will cause a significant redistribution of the locked-in stresses, highlighting the complexity of managing these stresses throughout the entire manufacturing chain [22].

### 1.2. Process-Induced Residual Stresses: Sources and Mechanisms

The presence of residual stresses is inextricably linked to the material’s microstructure, as both are consequences of the same thermomechanical history imposed during manufacturing [23,24]. The thermal cycles and plastic deformation that generate residual stresses are the very same mechanisms that drive microstructural evolution, including phase transformations, grain size and morphology changes, and the generation and arrangement of dislocations [25,26,27]. In additive manufacturing, for example, the rapid solidification and repeated heating and cooling cycles create unique and often anisotropic microstructures, such as elongated columnar grains, which are directly associated with the high internal stresses characteristic of these processes [28]. This combined state of microstructure and residual stress ultimately dictates the final mechanical performance of the component, including its hardness, yield strength, and ductility [24,29]. For instance, the evolution of strengthening precipitates and grain structure in high-strength aluminum alloys is directly tied to the development of residual stress during quenching [30]. Consequently, this relationship can be intentionally manipulated through processing. Post-fabrication heat treatments are often employed specifically to modify the microstructure, for example, to induce recovery and recrystallization, with the express purpose of relieving residual stresses and improving mechanical behavior [31]. Similarly, mechanical pre-treatments can be used to engineer a desired initial microstructure and stress state [32]. Therefore, quantitatively evaluating these interactions, such as by correlating dislocation density with the magnitude of residual stress, is a critical area of research for achieving predictive control over material performance.

To understand, predict, and control residual stresses, the field relies on a combination of sophisticated computational modeling and precise experimental measurement techniques. Modeling and simulation, predominantly executed through the finite element method (FEM), have become indispensable tools for predicting the evolution of residual stress and distortion during manufacturing processes [33,34]. These physics-based models simulate the complex thermomechanical phenomena, allowing for virtual testing and process optimization before physical production begins. Furthermore, a key emerging trend is the enhancement of these simulations by integrating them with experimental data, creating hybrid approaches that significantly improve predictive accuracy [35]. However, experimental validation and direct characterization remain crucial, and for this, a diverse array of measurement techniques is available, broadly categorized as non-destructive, semi-destructive, and destructive [36]. Non-destructive techniques are highly valued for quality control as they do not damage the component. Among these, diffraction-based methods such as X-ray diffraction (XRD) for surface measurements and neutron diffraction for bulk measurements are widely used and considered standards in the field [37]. Other non-destructive approaches, including ultrasonic and magnetic methods, are also an active area of research [38]. For higher-resolution or through-thickness measurements, semi-destructive methods like hole drilling and the contour method are often employed, providing detailed stress profiles at the cost of minor, localized damage to the part. The selection of the most appropriate technique is a complex decision that depends on factors such as the material, the required measurement depth and resolution, and the geometry of the component, often necessitating a combination of simulation and multiple measurement methods for a comprehensive analysis [39].

Additionally, the sign, magnitude, and distribution of residual stresses have a profound impact on the structural integrity and service life of metallic components, directly influencing a variety of failure mechanisms [40]. One of the most immediate and detrimental effects of uncontrolled residual stress is geometrical distortion and a loss of dimensional stability, which is a primary concern in precision manufacturing processes such as welding [41]. Beyond dimensional inaccuracies, residual stresses are a critical factor in the material’s resistance to fatigue failure. Tensile residual stresses are particularly damaging as they effectively increase the mean stress of a cyclic load, thereby lowering the fatigue limit and accelerating crack initiation and growth. Conversely, intentionally introducing deep, stable layers of compressive residual stress through surface treatments like shot peening is a widely used and highly effective strategy for enhancing fatigue life and resistance to corrosion fatigue [42]. The interplay between residual stress and environmental degradation is another critical area of concern. High tensile residual stresses can significantly increase a material’s susceptibility to stress corrosion cracking (SCC) and other forms of corrosion [43,44]. Furthermore, the relaxation of beneficial compressive stresses over time can compromise the long-term corrosion fatigue performance of a component. This influence extends to even more complex, synergistic failure modes like tribocorrosion, where the combined effects of mechanical wear and chemical corrosion are also heavily dependent on the underlying residual stress state [45]. Therefore, as mentioned by Minamizawa et al. [46], managing residual stress is not merely about preventing initial defects but is fundamental to controlling a component’s long-term durability and resistance to a host of complex degradation phenomena.

### 1.3. Scientific Challenges and Rationale for a Bibliometric Review

Therefore, given the complexity and rapid growth of research into residual stress, state-of-the-art reviews and meta-analyses are indispensable for consolidating knowledge, identifying challenges, and charting future research directions [47,48]. A survey of recent literature reviews reveals a significant and sustained focus on the challenges within AM, where numerous studies have synthesized the current understanding of the evolution, control, mitigation, and evaluation of residual stresses in additively manufactured metallic parts [49,50,51]. Concurrently, reviews on conventional processes like welding and machining highlight a push towards more advanced predictive models, including machine learning approaches, and a continued focus on process optimization [47]. Another prominent trend identified in recent overviews is the advancement of measurement techniques, with focused reviews on emerging methods like instrumented indentation and comprehensive summaries of the state of the art in characterization [52,53]. Critically, these review articles serve a purpose far beyond simple summarization. They are essential scholarly tools that synthesize vast amounts of disparate data, allowing the community to distinguish established principles from unresolved questions and identify persistent research gaps. By mapping the current landscape, they provide a strategic framework that helps guide future investigations toward the most pressing challenges, such as the widely recognized need for better in-process monitoring and more accurate predictive models. Thus, these high-level analyses provide the necessary perspective to understand the trajectory of a complex scientific field, making them invaluable for directing efficient and impactful research. This underscores the motivation for the present study, which aims to provide a broad, quantitative overview of the entire research domain through bibliometric analysis.

Building upon the established value of such systematic overviews, the primary objective of this paper is to conduct a comprehensive bibliometric analysis to quantitatively map the intellectual structure and evolution of research on residual stresses in metal manufacturing. This study first details the methodology employed for data acquisition from the Scopus and Web of Science databases and the analytical tools used for network visualization and analysis. Subsequently, the results are presented, focusing on publication trends over time, the most influential countries and institutions, keyword co-occurrence networks, and the emergence of dominant thematic clusters. By providing a holistic and data-driven perspective on the field, this work aims to identify the principal research fronts, uncover existing knowledge gaps, and highlight promising avenues for future investigation, thereby serving as a valuable resource for researchers, engineers, and practitioners.

## 2. Methods

**Figure 1:**
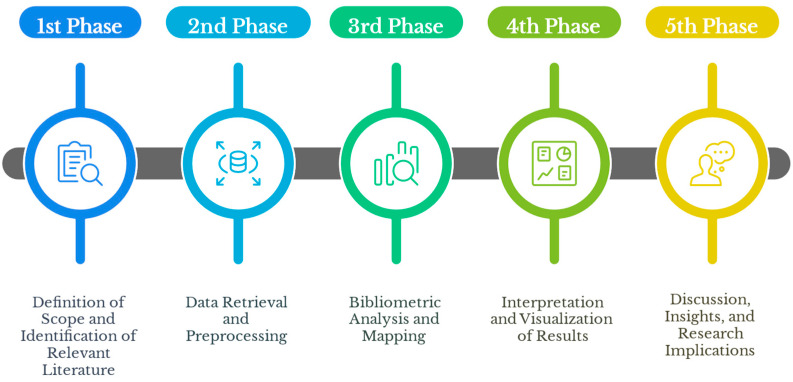
Schematic representation of the bibliometric review methodology applied in this study, structured into five sequential phases: (i) definition of objectives and identification of literature, (ii) data retrieval and preprocessing, (iii) bibliometric mapping using Bibliometrix and Biblioshiny, (iv) interpretation and consolidation of results, and (v) discussion of implications for science and industry.

This bibliometric study was conducted following a systematic methodology, which structures the process into five distinct and sequential phases. Each phase is represented in Figure 1, which outlines the overall workflow adopted for this review. The phases include: (i) definition of objectives and identification of relevant literature, (ii) data retrieval and preprocessing, (iii) bibliometric mapping, (iv) interpretation and consolidation of results, and (v) discussion of the impact and implications of the findings. This stepwise approach ensures methodological transparency, reproducibility, and a clear path from data acquisition to interpretation.

**Figure 2:**
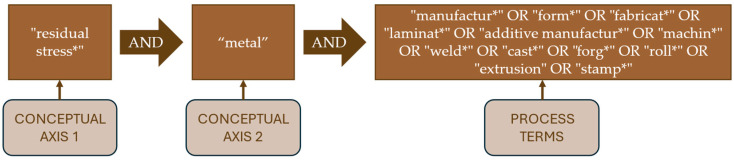
Search strategy matrix used to construct the bibliographic dataset. The matrix combines three conceptual axes: residual stress-related terms (Axis 1), material focus on metals (Axis 2), and manufacturing processes (Axis 3). Boolean operators and wildcards (*) were used to expand the scope and ensure comprehensive retrieval.

In the first phase, the scope of the study was defined, and the main axes of analysis were established. This involved identifying the core concepts and technical terminology associated with residual stresses in the context of metal manufacturing. As part of this stage, a search strategy was developed to encompass both the phenomena under investigation (residual stresses) and the materials and processes involved. To ensure a comprehensive literature search, a search string was developed using a keyword matrix that combined conceptual and process-related terms, as detailed in Figure 2. The search terms were grouped into three key components: (i) Conceptual Axis 1, where the primary scientific concept was “residual stress *”, with the asterisk (*) ensuring the inclusion of both singular and plural forms, as well as potential derivations; (ii) Conceptual Axis 2, where the material focus was defined using the term “metal *”, which allows for retrieval of the literature referring to metals, metallic components, or metal-based systems, following the asterisk strategy; and (iii) Process Terms, referring to the operations in which residual stresses commonly occur, such as manufacturing, forming, fabrication, lamination, machining, welding, casting, forging, rolling, extrusion, and stamping, which have been included. Here, by using Boolean operators (AND, OR), the search string was constructed to intersect the conceptual axes with relevant manufacturing processes. This method allowed us to retrieve studies that explicitly address residual stress within metallic systems subjected to various fabrication or forming techniques.

In the second phase of the study, a systematic and reproducible literature search was conducted across two of the most prestigious and widely used bibliographic databases: Web of Science (WoS) and Scopus. These platforms were selected due to their extensive multidisciplinary coverage, rigorous indexing protocols, and predominant inclusion of peer-reviewed scientific publications [54,55]. The bibliometric analysis was limited to a five-year time span, ranging from 2019 to 2024, in order to focus on the most recent developments and research trends in the field. As a result of the initial query—developed using a structured Boolean search strategy (see Figure 2)—a total of 6662 records were retrieved: 3172 from WoS and 3490 from Scopus. This raw dataset represented a diverse and extensive body of literature encompassing multiple scientific fields, publication years, and types of documents. At this point, it is important to note that methodological transparency and replicability were ensured, as the entire screening and selection process strictly adhered to the PRISMA 2020 guidelines (Preferred Reporting Items for Systematic Reviews and Meta-Analyses). The PRISMA 2020 statement provides a standardized framework to improve the clarity, consistency, and reproducibility of literature reviews, especially those involving large datasets, such as bibliometric studies [56,57]. By following this protocol, the study ensures that the identification, selection, and inclusion of sources are systematic and bias-minimized.

**Figure 3:**
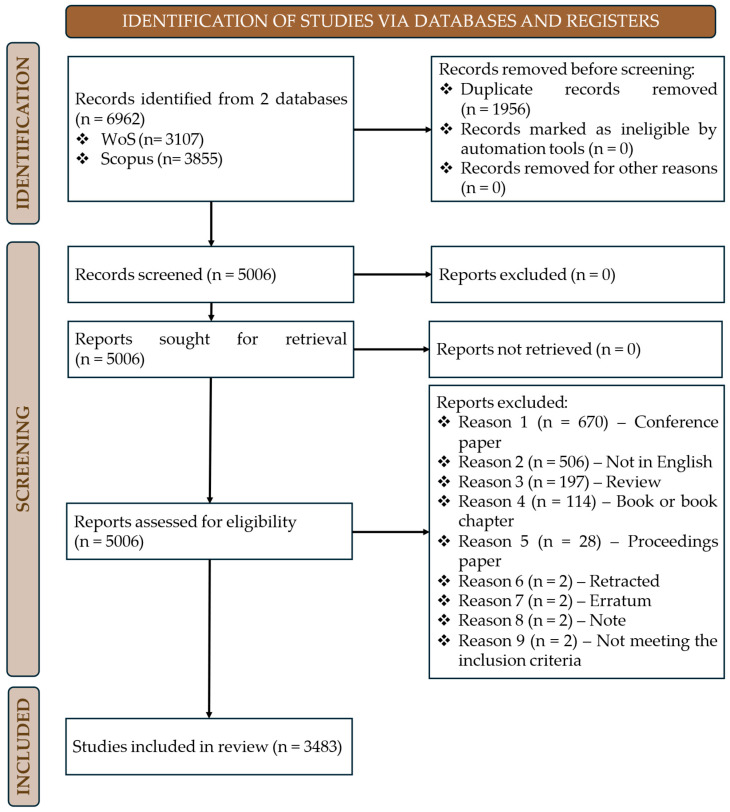
PRISMA 2020 flow diagram illustrating the literature screening and selection process. The diagram outlines the number of records identified from Scopus and Web of Science, duplicates removed, documents excluded by type or language, and the final dataset of 3483 articles included for bibliometric analysis.

As illustrated in Figure 3, the identification phase began with the export and merging of all records retrieved from both databases. Following this, a total of 1596 duplicate records were identified and removed, resulting in 5066 unique entries. During the screening phase, all records were evaluated based on their titles and abstracts to determine thematic alignment with the core topic of residual stresses in metal manufacturing. A rigorous filtering procedure was implemented to refine the dataset to include only peer-reviewed scientific articles published in English. Here, a total of 1523 records were excluded, including 670 records identified as conference papers, 506 publications not written in English, 197 review articles, 114 book or book chapter entries, and 28 proceedings papers, in addition to 2 records excluded for each of the following reasons: retracted publications, errata, and notes. Finally, two studies were removed for not meeting the established inclusion criteria. It is noteworthy that no records were removed due to automation-related screening, algorithmic ineligibility, or technical retrieval issues, thereby ensuring the transparency, reliability, and reproducibility of the data collection process. Upon completion of this comprehensive screening phase, a final set of 3483 articles—published between 2019 and 2024—was retained as the core corpus for bibliometric analysis.

In the third phase, bibliometric mapping was carried out using the Bibliometrix R-4.5-0 package, a comprehensive open-source tool developed for quantitative research in scientometrics and bibliometrics [58]. To facilitate a more intuitive interaction with the dataset and to generate dynamic visualizations, the Biblioshiny web interface—a Shiny app integrated within Bibliometrix—was employed. This allowed for the generation of data and impact plots.

The fourth phase involved the interpretation and consolidation of the bibliometric findings, with a particular focus on understanding the structural and thematic dimensions of the field. Through co-word analysis, thematic clustering, citation metrics (e.g., h-index, g-index, and total citations), etc., the integration of multiple indicators enabled a more nuanced and multidimensional understanding of the scientific landscape.

Finally, in the fifth phase, the results were critically analyzed to discuss the impact and implications of the findings for both the academic and industrial communities. Special attention was given to identifying underexplored areas, interdisciplinary intersections, and technological challenges associated with residual stress analysis and control in metal manufacturing.

## 3. Results

The results are organized to provide a comprehensive view of the research landscape, including descriptive statistics, journal productivity and impact, institutional and national contributions, influential publications, keyword analysis, thematic evolution, and process-specific research focus. Through these indicators, the study captures both the structural composition and emerging trends of the field.

### 3.1. Descriptive Overview of the Bibliographic Dataset

**Figure 4:**
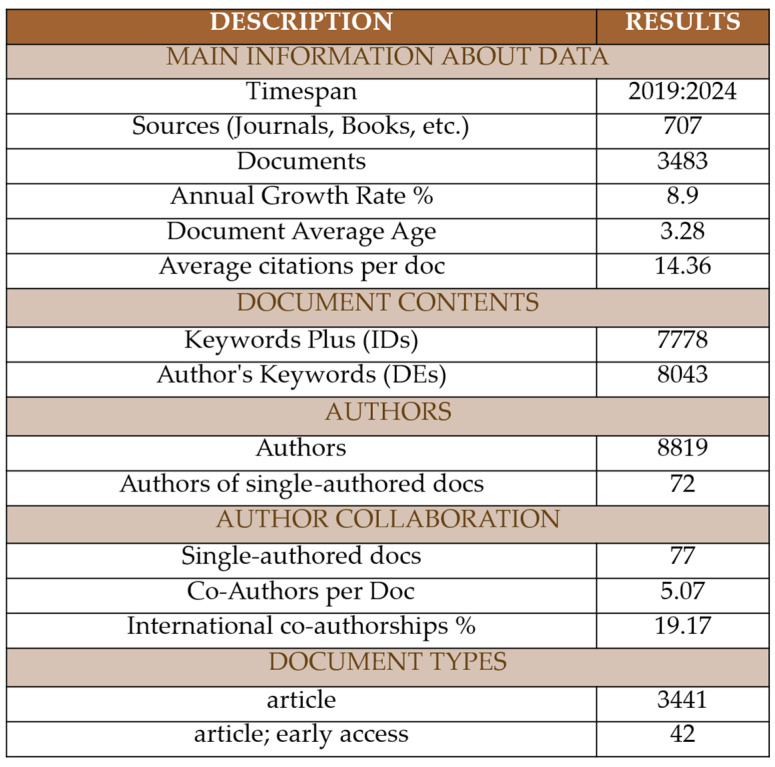
Summary of descriptive bibliometric indicators for the final dataset (2019–2024), including number of articles, sources, authors, collaboration metrics (e.g., co-authorship index), keyword occurrences, document types, and average citation metrics.

This section provides a concise overview of the bibliographic dataset comprising 3483 peer-reviewed publications on residual stress in metal manufacturing from 2019 to 2024. The bibliometric performance and descriptive metrics of this dataset are presented in Figure 4. These documents originated from 707 distinct sources, all corresponding to peer-reviewed journals, in accordance with the criteria detailed in the Methods section. The field has exhibited an average annual growth rate of 8.9%, reflecting a consistent and sustained research interest.

In terms of authorship, 8819 unique researchers contributed to the publications. Although only 77 documents were authored individually, 72 distinct authors were responsible for these single-authored works. The average number of co-authors per paper was 5.07, indicating a moderate to high degree of collaboration within the research community. Furthermore, 19.17% of the publications involved international co-authorships, underscoring the global relevance of residual stress research in both academic and industrial contexts.

Regarding content, the dataset included 7778 Keywords Plus—terms automatically generated from cited references, also referred to as IDs (Identifier Keywords)—and 8043 Author’s Keywords—manually provided by the authors themselves, also known as DEs (Descriptive Keywords). This lexical richness provides a robust foundation for subsequent co-word and thematic analyses. As expected in a technically focused and applied research area, the predominant document type was the original research article, with a smaller share represented by early access articles. At this point, it is important to note that all the documents were peer-reviewed scientific papers, as indicated by the filtering criteria shown in Figure 3. The dataset also revealed an average document age of 3.28 years and an average of 14.36 citations per document, indicating a growing impact and increasing visibility of recent studies in the field, especially considering that the analysis covers only the last six years.

**Figure 5:**
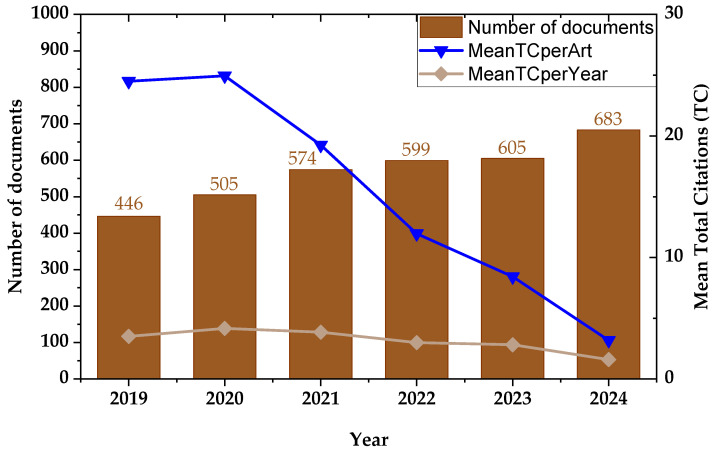
Annual scientific production and citation impact of residual stress research in metal manufacturing from 2019 to 2024. Brown bars represent the number of publications per year. Blue and beige lines show the mean total citations per article and per year, respectively, revealing a temporal trend in research visibility.

Figure 5 shows the annual evolution of scientific output and citation performance in the area. The brown bars represent the number of documents published each year, showing a steady increase from 446 publications in 2019 to 683 in 2024, which reflects a clear upward trend and growing research interest in the topic. Overlaid on the bar chart, the blue line plots the average number of total citations per article (Mean TC per Article), while the beige line represents the average total citations received per year (Mean TC per Year). Although the number of publications increased annually, the mean citations per article and per year displayed a declining trend, particularly after 2020. This pattern suggests that newer publications have not yet had sufficient time to accumulate citations at the same rate as earlier works—a common phenomenon in rapidly evolving fields, where the pace of publication outstrips the time required for citation maturation. Nonetheless, the consistent growth in publication output underscores the expanding scientific attention and relevance of the field.

### 3.2. Analysis of Core and Impactful Journals

A comprehensive analysis of the most relevant scientific journals was conducted to identify both the most productive sources and the most impactful outlets in the domain of residual stress research in metal manufacturing. This subsection presents the core journals as determined by Bradford’s Law and evaluates their scholarly influence through citation-based indicators and trends in scientific output. It is important to emphasize that the identification of core journals in Table 1 is based on Bradford’s Law, which prioritizes publication volume within the specific scope of residual stress in metal manufacturing. This approach does not necessarily reflect the broader scientific impact or prestige of each journal, but rather their relative productivity in the selected dataset.

Table 1 shows the ranking of journals based on productivity. According to Bradford’s Law, scientific literature in a specific field tends to be distributed unevenly across journals; a small number of journals (core or Zone 1) publish most relevant articles, followed by a larger set of journals with moderate productivity (Zone 2) and a final, even larger group with low productivity (Zone 3). According to Bradford’s Law, nine journals form the core zone (Zone 1), concentrating the highest number of publications on residual stresses in metal manufacturing. *Materials* stands out as the most prolific journal with 171 publications, followed by *International Journal of Advanced Manufacturing Technology* (151) and *Metals* (114). Other significant contributors include *Journal of Manufacturing Processes*, *Journal of Materials Engineering and Performance*, and *Additive Manufacturing*. These journals primarily focus on advanced manufacturing, materials science, and process optimization.

In this line of study, Table 2 highlights the most influential journals based on bibliometric indicators such as the h-index, g-index, and m-index. These indicators offer complementary views of scholarly impact: the h-index reflects the number of publications (h) that have received at least h citations each; the g-index gives more weight to highly cited articles, representing the highest number (g) such that the top g articles have at least g^2^ cumulative citations; and the m-index adjusts the h-index by the number of years since the first relevant publication, thus accounting for career or journal “age”. Thus, additive manufacturing leads in impact, with the highest h-index (37), g-index (58), and total citations (4014), suggesting a high level of scientific influence despite its relatively recent indexation in 2019. Other notable journals include *Journal of Manufacturing Processes* and *Materials Science and Engineering A*, which also show strong citation metrics, reflecting their sustained contributions to the field. Notably, many of these journals began indexing relevant articles after 2019, indicating a recent surge in interest and scientific output in this domain. It is important to note that the scholarly impact indicators presented in Table 2 are computed specifically within the retrieved dataset focused on residual stress in metal manufacturing; as a result, some globally prestigious journals may not appear in this ranking due to a relatively lower number of topic-specific articles during the analyzed period.

**Figure 6:**
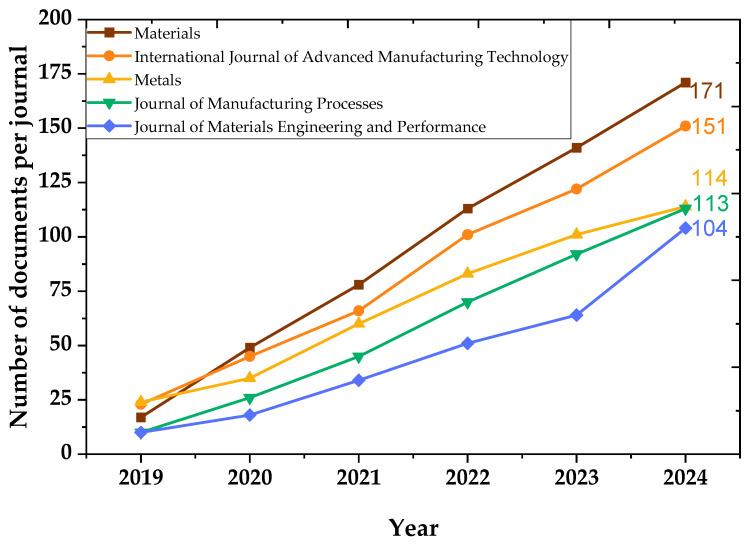
Temporal evolution of journal productivity (2019–2024).

Figure 6 presents the annual number of publications from the five most prolific journals between 2019 and 2024. *Materials* exhibits the most significant growth, peaking at 171 documents in 2024. In addition, *International Journal of Advanced Manufacturing Technology and Metals* has shown sustained increases in scientific output, indicating a robust and expanding research environment. *Journal of Manufacturing Processes* and *Journal of Materials Engineering and Performance* also reflect consistent growth, though at a slightly lower rate. This trend confirms a growing research focus on residual stresses, closely aligned with the evolution of advanced manufacturing technologies. The rising number of documents per year underscores the field’s maturation and the increasing complexity of manufacturing challenges that require detailed stress analysis and control.

### 3.3. Institutional and National Contributions

This section analyzes the institutional and national landscape of research on residual stresses in metal manufacturing. It highlights the most productive institutions, their growth trends, and the global distribution and impact of scientific output, offering insight into key contributors and emerging leaders in the field.

**Figure 7:**
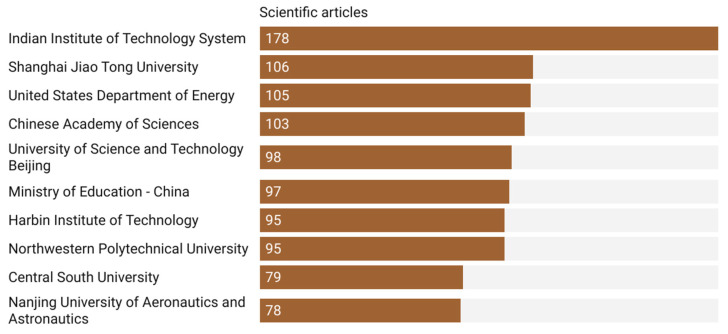
Top contributing institutions based on the number of publications in the domain of residual stresses in metal manufacturing (2019–2024).

Figure 7 illustrates the leading institutions in terms of total number of publications. The Indian Institute of Technology System (IITS) clearly dominates the field with 178 articles, significantly surpassing Shanghai Jiao Tong University (106) and the United States Department of Energy (105). The presence of three Chinese institutions (Shanghai Jiao Tong University, University of Science and Technology Beijing, and the Chinese Academy of Sciences) and one Indian institution in the top five emphasizes the prominent role of Asia in driving research in this domain. It is also notable that several of these institutions are either directly supported by national governments or play a strategic role in national technological development, such as the Ministry of Education for China and the US Department of Energy.

**Figure 8:**
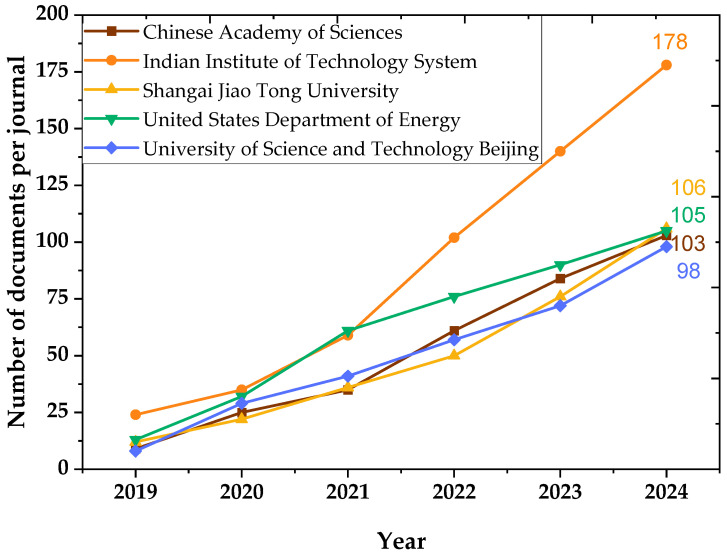
Temporal evolution of scientific output from leading institutions (2019–2024).

Figure 8 tracks the growth in scientific production from the top five institutions over time (2019–2024). The Indian Institute of Technology System displays the most significant upward trend, almost doubling its output each year. The Chinese Academy of Sciences follows a similar trajectory, underscoring China’s expanding investment in materials science. The steady increases seen across all institutions suggest that the topic of residual stress is not only gaining scientific relevance but also institutional and industrial priority in key technological nations.

**Figure 9:**
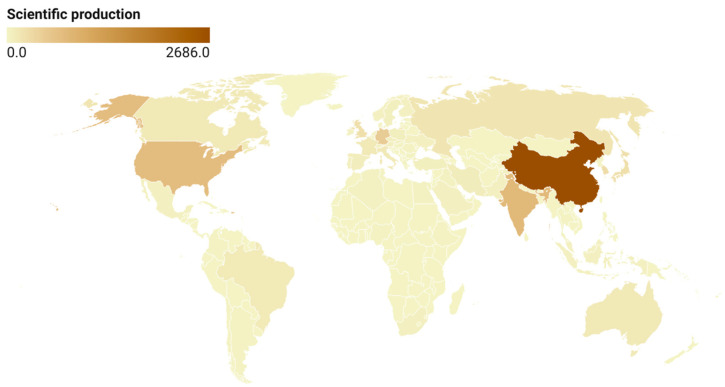
Global scientific output (2019–2024).

Figure 9 shows a global heatmap of scientific output, with darker regions indicating higher productivity. China stands out as the most prolific contributor, followed by the United States and India. This distribution reflects regional research strengths in metal manufacturing, particularly in Asia–Pacific and North America. The emergence of China as a global research leader is aligned with its broader strategic goals in advanced manufacturing and smart materials.

Table 3 complements the production data with citation impact, revealing interesting contrasts between quantity and quality. China leads in total citations (16,409), but with a relatively modest average of 13.8 citations per article, suggesting a high volume of output with moderate per-paper influence. In contrast, Singapore exhibits a remarkably high average citation rate of 55.2 despite a relatively small number of publications (1326 citations), indicating that its contributions, though fewer, are particularly impactful. The United Kingdom (27.0), United States (22.7), and Italy (22.0) also demonstrate strong citation performance, balancing volume with scholarly visibility. These patterns suggest that while China is the dominant contributor in terms of quantity, Western countries often produce work that garners broader academic recognition.

### 3.4. Most Influential Scientific Articles

In this section, the top ten most cited articles in the field are stated (Table 4). The article by David Svetlizky et al. [59] stands out with 644 total citations and the highest annual citation rate (128.8), indicating exceptional visibility and influence. João Pedro Oliveira et al. [60]. and Bassiouny Saleh et al. [61] follow with 564 and 456 citations, respectively. Normalized citation values also emphasize the relative impact of these works. Notably, recent articles such as those by Jin Fu et al. [62] and Jincheng Wang et al. [63], despite their recent publication dates (2022 and 2023), exhibit high normalized citation scores (18.48 and 23.03), underscoring their rapid scientific uptake. These results reveal that high-impact contributions are not limited to older publications and that cutting-edge studies in this area are being rapidly incorporated into scientific discourse.

## 4. Discussion

This section analyzes the results of the bibliometric analysis, highlighting the most influential trends, topics, and contributors in the field. By examining citation patterns, keyword dynamics, thematic structures, and process-specific distributions, the discussion provides insights into how the scientific community is addressing significant challenges and advancing knowledge in this area. The analysis also identifies emerging areas of interest and suggests future research opportunities aligned with technological developments and industrial needs.

### 4.1. Analysis of the Most Impactful Documents and Citation Trends

The top ten most cited articles in the domain of residual stress in metal manufacturing reflect the field’s evolution through four principal research trends: (i) process optimization in additive manufacturing (AM), (ii) surface integrity in subtractive and hybrid processes, (iii) advanced materials and functionally graded systems, and (iv) defects and mechanical performance. These trends demonstrate the diversification of manufacturing technologies and the importance of understanding the origins, control, and implications of residual stress across different processing conditions and material systems.

The first trend observed among the most influential publications is the emphasis on process optimization in AM. Specifically, a dominant focus lies in the control of residual stresses through the fine-tuning of process parameters, highlighting the critical role of parameter selection in minimizing stress-related defects and improving component performance. Svetlizky et al. [59] provide a comprehensive review of directed energy deposition, analyzing key thermal cycles, melt pool size, and cooling rates. They note that stress magnitudes in Ti-6Al-4V components can exceed yield strength when insufficient cooling is applied, highlighting the necessity of optimized interpass temperatures. Oliveira et al. [60] study laser powder bed fusion of 316 L stainless steel, showing that a combination of reduced laser power (100–150 W) and increased scan speed can significantly decrease residual tensile stress by reducing thermal gradients and promoting uniform solidification. Similarly, Jafari et al. [64] focus on wire arc AM with ER70S-6 wire, demonstrating that an inter-layer dwell time of over 60 s reduces tensile stress by more than 30% via partial annealing. Wang et al. [63] use high-speed imaging and X-ray diffraction to quantify stress-induced porosity, finding a strong correlation between melt pool instability and pore clustering. These studies demonstrate that AM residual stress is a multifactorial outcome driven by energy input, path strategy, and thermal management. These parameters require fine-tuning for process reliability and structural performance.

The second trend highlights surface integrity in subtractive and hybrid processes, where residual stresses arise from mechanical and thermal effects, emphasizing the need to mitigate their impact on part quality. Liao et al. [65] evaluate machining-induced stress in hardened steels and titanium alloys, detailing how cutting speed, feed rate, and tool geometry influence the depth and gradient of tensile stress layers. Their findings suggest that finishing processes using low cutting speeds (below 50 m/min) and optimized tool angles can reduce surface tensile stress, delaying crack initiation. Tong et al. [66] conduct an in-depth study on Al-Cu-Li alloys, showing how solid-solution treatments followed by artificial aging influence dislocation recovery and stress relaxation, reducing residual stress by up to 40%. This underscores the importance of both mechanical and thermal contributions to stress formation in post-AM or conventionally fabricated parts, suggesting a hybrid paradigm for stress management.

The third trend involves advanced materials and functionally graded systems, where material design plays a critical role in stress development, as demonstrated in studies on thermally sensitive alloys and graded compositions. Saleh et al. [61] review the fabrication of functionally graded materials via powder metallurgy and laser cladding, showing that residual stress tends to concentrate at abrupt composition gradients, especially between metal and ceramic phases. They highlight that gradual transitions and layer preheating are effective strategies to reduce interfacial stress. Yakout et al. [68] provide experimental data from L-PBF processing of Invar 36 and 316 L, concluding that a volumetric energy density of ~60 J/mm^3^ minimizes both porosity and residual stress. Their stress measurement via the contour method showed reductions of up to 50 MPa compared with higher energy input conditions. These studies reinforce that compositional design must be accompanied by thermal control strategies to mitigate stress in complex, multi-phase builds.

A final but critical trend involves linking residual stress to defect formation and mechanical degradation. Fu et al. [62] propose a classification of AM defects, correlating residual stress with surface microcracks and internal lack-of-fusion defects. Using electron backscatter diffraction and synchrotron X-ray tomography, they reveal that stress concentrations exceeding 300 MPa can cause crack propagation along melt track boundaries. Becker et al. [67] investigate fracture toughness in selective laser melting-processed IN718, identifying anisotropic grain morphology and residual stress as primary contributors to intergranular failure. They show that stress relief heat treatment reduces crack density by nearly 60%, improving fatigue life under cyclic loading. Again, Wang et al. [63] support these observations by connecting melt pool dynamics with porosity-induced stress concentrations, which serve as nucleation sites for failure. The convergence of these works highlights residual stress as an active factor in material degradation, underscoring the need for integrated defect engineering strategies.

To sum up this section, the most cited publications position residual stress as a key factor linking process design, material behavior, and performance. Their high impact reflects the field’s recognition of stress control as essential for advancing precision and structural integrity in metal manufacturing.

### 4.2. Analysis of Most Frequent Keywords and Word Analysis

This section provides an in-depth examination of the most frequent keywords and their interconnections within the scientific literature on residual stress in metallic manufacturing. The analysis of keywords reveals the core concepts and underlying structure of knowledge production in the field. It is important to note that some terms, particularly those related to manufacturing processes, appear frequently due to their inclusion in the search string used to construct the dataset.

Table 5 presents the frequency of keywords extracted from the dataset. Here, the most recurrent term, residual stress, appeared 1513 times, affirming its central role and justifying the thematic scope of the bibliometric analysis. This high frequency confirms that the quantification, modeling, and mitigation of residual stresses are critical research concerns across metal manufacturing domains. The second and third most frequent terms—microstructure (772 occurrences) and mechanical-properties (565)—underscore the material science-driven approach of much of the literature, where the microstructural consequences of residual stresses are tightly interlinked with mechanical performance indicators such as strength (237) and hardness (88) [24,31,69]. Various studies have explored the role of residual stresses in metal manufacturing, highlighting their critical influence on mechanical properties, dimensional stability, and structural integrity. Research has investigated the effect of different welding techniques on stress distribution, the use of post-processing heat treatments to relieve stresses in AM components, and the application of optical methods for non-destructive residual stress characterization [69,70,71].

Additionally, keywords such as simulation (236), model (183), and parameters (174) indicate a robust computational and parametric modeling trend, which supports the development of predictive frameworks for residual stress evolution. These terms align with finite element modeling (FEM) and thermomechanical simulations commonly applied in both academic and industrial contexts. Moreover, the integration of FEM with experimental validation has further enhanced its credibility as a predictive approach for stress analysis in complex geometries and material systems [8,72,73].

The appearance of specific materials such as steel (169) [74,75], titanium (96) [76], and Ti-6Al-4V (113) [77,78] points to application-driven studies, often related to aerospace, biomedical, or high-performance manufacturing. Other significant terms such as deposition, laser, and process parameters reveal the increasing integration of AM and laser-based processes in this research domain, where control over residual stresses is pivotal for component integrity and service life.

In this regard, semantic analysis of the keywords reveals that certain terms related to specific types of metals—such as steel, titanium, stainless steel, alloy, Ti-6Al-4V, aluminum, and nickel—appear with remarkable frequency, indicating a strong focus on studies oriented towards specific materials. This distribution suggests that the nature and magnitude of residual stress are strongly influenced by the chemical composition, thermal conductivity, thermal expansion coefficient, and phase transformations specific to each metal or alloy. For example, in steels, especially martensitic or heat-treated steels, residual stresses commonly arise from phase transformations such as austenite-martensite, with critical effects on fracture toughness and fatigue resistance. In contrast, titanium alloys such as Ti-6Al-4V show a high propensity to develop residual tensile stresses during additive manufacturing due to their low thermal conductivity and high temperature gradients, requiring specific scanning, preheating, or thermal relief strategies. Aluminum alloys, on the other hand, although less prone to generating severe internal stresses thanks to their good thermal conductivity, often suffer distortions during subsequent machining processes due to stresses introduced during cooling or forming. Finally, in nickel superalloys, widely used in high-temperature applications, the complexity of precipitation hardening mechanisms and the multiple phases present mean that the control and characterization of internal stresses require advanced methods such as neutron diffraction or synchrotron tomography. Thus, this analysis shows that not only manufacturing processes, but also the metallurgical nature of the material, profoundly condition the appearance, evolution, and consequences of residual stresses, justifying the need for differentiated approaches by metal class in future research.

**Figure 10:**
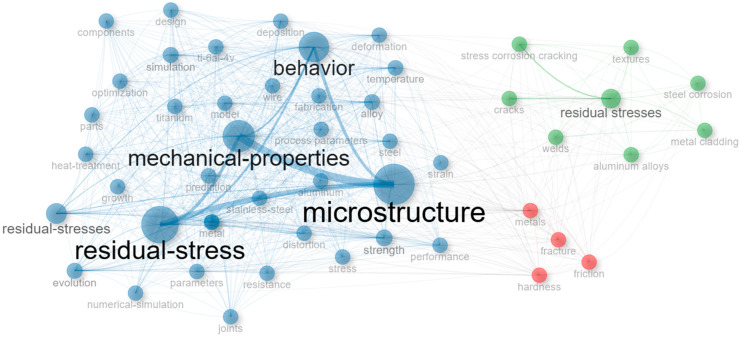
Keyword co-occurrence network generated from the Author’s Keywords dataset. Node size represents keyword frequency, while proximity and connection strength reflect co-occurrence relationships. Central clusters include key terms such as residual stress, microstructure, mechanical properties, simulation, and heat treatment.

Figure 10 depicts a semantic co-occurrence network of keywords, visualizing the conceptual system of residual stress research. The size of each node reflects its frequency, while the proximity and linkage strength between nodes indicate their semantic closeness based on co-appearance in scientific documents.

The largest and most central nodes—residual-stress, microstructure, and mechanical-properties—form the conceptual nucleus of the field. These keywords are connected to subdomains related to simulation and physical modeling (model, temperature, distortion, simulation), as well as to performance-oriented concepts such as behavior, performance, and resistance. This network structure highlights how the field balances physical process understanding with performance optimization, as stated in the literature [79,80,81].

The strong association between microstructure and terms like heat-treatment, strength, and performance points to a well-established research thread focused on how residual stresses alter microstructural evolution and, by extension, influence material reliability and service performance. Such relationships are critical in high-stakes applications like aerospace or power generation, where stress-related degradation can lead to premature failure [82,83].

A peripheral cluster in the network—comprising stress corrosion cracking, cracks, and hardness—indicates a growing body of work examining damage mechanisms that are either induced or aggravated by residual stresses [84,85]. This suggests an emerging focus on fracture mechanics and environmental degradation, extending the research frontier toward long-term durability assessments.

### 4.3. Thematic Structure and Strategic Diagram

**Figure 11:**
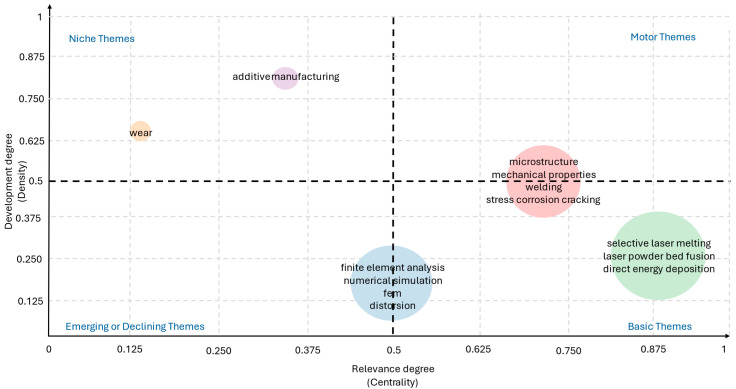
Strategic thematic map derived from the co-word analysis. Themes are plotted according to their centrality (horizontal axis: relevance to the field) and density (vertical axis: internal development). Quadrants represent Motor Themes (**top right**), Basic Themes (**bottom right**), Niche Themes (**top left**), and Emerging or Declining Themes (**bottom left**).

Figure 11 presents the thematic map generated through co-word analysis, which provides a strategic visualization of the conceptual scenery in the field. The map plots clusters of keywords along two axes: centrality (horizontal) and density (vertical), which indicate the theme’s relevance within the overall research field and its level of internal development, respectively. Axes are normalized from 0 to 1, with each subdivision representing an increment of 0.125. The *X*-axis (Relevance degree) indicates the centrality of each theme within the global research field, while the *Y*-axis (Development degree) reflects the internal density and maturity of each thematic cluster.

The upper-right quadrant, labeled Motor Themes, contains well-developed and central topics that are crucial for the structure of the research field. Here, we find clusters associated with microstructure, mechanical properties, welding, and stress corrosion cracking. These terms suggest a strong and mature research focus on the relationship between processing conditions and resulting mechanical performance in metallic components.

The lower-right quadrant, Basic Themes, includes concepts such as selective laser melting, laser powder bed fusion, and direct energy deposition. These are foundational yet relatively underdeveloped topics in terms of thematic cohesion. Their high centrality implies that they are frequently cited or used across different studies, forming part of the essential knowledge base, particularly in the context of additive manufacturing technologies.

The upper-left quadrant, Niche Themes, contains more specialized or isolated lines of research, such as additive manufacturing and wear. These themes exhibit high density but lower centrality, meaning they are well developed within themselves but less connected to other research clusters. Their appearance in this quadrant suggests a concentration of detailed investigations that, while technically advanced, remain peripheral in terms of broader field influence.

Finally, the lower-left quadrant, Emerging or Declining Themes, includes finite element analysis, numerical simulation, distortion, and FEA. The low centrality and density values suggest these topics are either emerging and still developing in relevance or are declining in research focus. However, considering the increasing importance of predictive modeling and process simulation in Industry 4.0, it is likely that these represent emerging rather than obsolete themes.

### 4.4. Dominant Processing Techniques

As stated during this paper, residual stress analysis in metallic components is highly influenced by the specific processing technique employed during manufacturing. To capture the distribution of research efforts across the most common fabrication methods, keyword frequency analysis was conducted based on the query refinements described in Table 6. These refinements allowed for disambiguation and more accurate clustering of studies relevant to each technique.

The results indicate that welding is the most extensively studied processing technique, representing 35.35% of the total records. This prominence aligns with welding’s widespread use in structural and industrial applications and the complex thermal gradients it induces, often leading to high residual stress accumulation. Additive manufacturing (AM) follows with 25.15%, reflecting the growing academic and industrial interest in this disruptive technology. The layer-by-layer deposition and thermal cycling inherent to AM processes significantly influence residual stress formation, making it a central topic in recent research. Machining, including operations such as milling and turning, accounts for 17.64%, reinforcing its critical role in surface integrity and final part performance. Though primarily subtractive, machining operations can introduce both tensile and compressive residual stresses depending on tool geometry, cutting speed, and material type.

Secondary techniques such as casting (5.46%), rolling (8.81%), and laminating (2.91%) also show relevant contributions, suggesting steady academic attention to traditional and hybrid processes. Meanwhile, forging, extrusion, and stamping exhibit lower representation, possibly due to lower perceived complexity or fewer experimental challenges in residual stress quantification.

In addition to the manufacturing process itself, the type and composition of the metal play a decisive role in the generation and evolution of residual stresses, as stated before. Each metal or alloy responds uniquely to thermal gradients, deformation rates, and solid-state transformations due to differences in thermal conductivity, expansion coefficients, and microstructural characteristics. For instance, titanium alloys such as Ti-6Al-4V, commonly used in additive manufacturing, are prone to residual tensile stresses because of their low thermal conductivity and high-temperature sensitivity. In contrast, steels—particularly martensitic grades—accumulate significant internal stresses during heat treatment due to volume changes associated with phase transformations. Aluminum alloys, though more thermally conductive, are susceptible to distortion in post-processing due to cooling-induced stress gradients. Meanwhile, nickel-based superalloys used in high-temperature environments present complex stress behaviors linked to precipitation hardening and require advanced control methods during both additive and subtractive manufacturing. These examples underscore the necessity of material-specific approaches in residual stress analysis, where the interaction between process parameters and metallurgical properties defines both stress distribution and mitigation strategies.

## 5. Conclusions

This bibliometric review analyzed 3483 peer-reviewed publications (2019–2024) on residual stresses in metal manufacturing, revealing a consistent annual growth rate of 5.93% and a clear rise in scientific interest. Four dominant research areas were identified: additive manufacturing optimization, surface integrity, advanced materials, and stress-induced defects.

Among manufacturing methods, welding, additive manufacturing, and machining lead the research, while simulation-based approaches—particularly FEM—and themes like microstructure, fatigue, and heat treatment form the intellectual core of the field.

Despite technological progress, challenges remain in standardizing measurement methods, improving predictive modeling, and enhancing process control. Future work should strengthen collaboration across academia, industry, and standardization bodies to address these gaps and promote reliable residual stress management in advanced metal manufacturing.

## Figures and Tables

**Table 1:** Core journals identified by Bradford’s Law (2019–2024).

Scientific Journal	Ranking	Frequency	Cumulative Frequency	Zone
*Materials*	1	171	171	Zone 1
*International Journal of Advanced Manufacturing Technology*	2	151	322	Zone 1
*Metals*	3	114	436	Zone 1
*Journal of Manufacturing Processes*	4	113	549	Zone 1
*Journal of Materials Engineering and Performance*	5	104	653	Zone 1
*Additive Manufacturing*	6	97	750	Zone 1
*Journal of Materials Research and Technology*	7	72	822	Zone 1
*Materials Science and Engineering A*	8	72	894	Zone 1
*Journal of Alloys and Compounds*	9	53	947	Zone 1
*Surface & Coatings Technology*	10	50	997	Zone 1

**Table 2:** Journals with the highest scholarly impact.

Scientific Journal	h-Index	g-Index	m-Index	Total Citations	Publications
*Additive Manufacturing*	37	58	5.286	4014	97
*Journal of Manufacturing Processes*	29	40	4.143	2248	113
*Materials Science and Engineering A*	28	45	4.000	2281	72
*International Journal of Advanced Manufacturing Technology*	21	35	3.000	1746	151
*Journal of Alloys and Compounds*	21	34	3.000	1328	53
*Materials*	21	35	3.000	2035	171
*Journal of Materials Processing Technology*	20	34	2.857	1266	45
*Surface & Coatings Technology*	20	31	2.857	1099	50
*Materials & Design*	19	45	2.714	2108	46
*Journal of Materials Research and Technology*	18	24	3.000	928	72

**Table 3:** Most cited countries.

Country	Total Citations	Average Article Citations
China	16,409	13.8
United States	6571	22.7
India	4212	11.7
Germany	2666	12.8
United Kingdom	2618	27.0
Italy	1496	22.0
Singapore	1326	55.2
Japan	1219	10.9
Australia	995	25.5
Canada	988	15.2

**Table 4:** Most cited articles ranked by total citations and citation impact indicators.

Reference	Authors	Total Citations	Total Citations per Year	Normalized Total Citations
[59]	David Svetlizky et al.	644	128.80	33.45
[60]	João Pedro Oliveira et al.	564	94.00	22.61
[61]	Bassiouny Saleh et al.	456	76.00	18.28
[64]	Davoud Jafari et al.	336	67.20	17.45
[65]	Zhirong Liao et al.	272	54.40	14.13
[66]	Zhaopeng Tong et al.	241	34.43	9.84
[67]	Thorsten Hermann Becker et al.	238	47.60	12.36
[62]	Jin Fu et al.	221	55.25	18.48
[68]	Mostafa Yakout et al.	199	28.43	8.12
[63]	Jincheng Wang et al.	194	64.67	23.03

**Table 5:** Most frequently occurring keywords (2019–2024).

Words	Occurrences
residual * stress *	1513
microstructure	772
mechanical-properties	565
strength	237
simulation	236
alloy	219
temperature	218
model	183
distortion	182
parameters	174
steel	169
deformation	164
laser	155
stainless-steel	146
deposition	119
performance	119
heat-treatment	116
stress corrosion cracking	116
ti-6al-4v	113
resistance	103
design	97
titanium	96
hardness	88
cracks	87
process parameters	84

* Word term variation.

**Table 6:** Distribution of publications by metal processing technique (2019–2024).

Processing Technique	Query Modification	Frequency	Percentage
Additive Manufacturing (AM)	AND (“additive manufactur *” OR “AM”)	968	25.12%
Welding	AND (“weld *” OR “welding”)	1360	35.35%
Machining	AND (“machin *” OR “milling” OR “turning”)	679	17.64%
Casting	AND (“cast *” OR “casting”)	210	5.46%
Forging	AND (“forg *” OR “forging”)	80	2.08%
Rolling	AND (“roll *” OR “rolling”)	339	8.81%
Extrusion	AND (“extrusion” OR “extrude *”)	63	1.64%
Stamping	AND (“stamp *” OR “stamping”)	36	0.94%
Laminating	AND (“laminat *” OR “lamination”)	112	2.91%

* Word variations and expansion of the search results.

## Data Availability

No new data were created or analyzed in this study. Data sharing is not applicable to this article.
